# Editorial: Advancing stem cell and extracellular vesicle therapies for autoimmune and cancer treatment

**DOI:** 10.3389/fimmu.2026.1947245

**Published:** 2026-08-07

**Authors:** Li Duan, Yujie Liang, Xiaobin Huang

**Affiliations:** 1 Department of Orthopedics, Shenzhen Second People’s Hospital, The First Affiliated Hospital of Shenzhen University, Shenzhen, Guangdong, China; 2 Engineering Research Center of Intelligent Rehabilitation, College of Rehabilitation Medicine, Jining Medical University, Jining, Shandong, China; 3 Department of Oral and Maxillofacial Surgery and Pharmacology, University of Pennsylvania, School of Dental Medicine, Philadelphia, PA, United States

**Keywords:** autoimmune disease, cancer, editorial, extracellular vesicles, stem cells

Translational medicine has increasingly turned its focus toward mesenchymal stem cells (MSCs) and MSC-derived extracellular vesicles (EVs), owing to their notable capacity to modulate immune responses with a favorable safety profile. Autoimmune diseases encompassing a broad spectrum of chronic conditions such as diabetes and arthritis pose substantial therapeutic hurdles, not least because their intricate interplay with tumor initiation and progression complicates clinical management, while conventional treatments frequently entail severe adverse effects. Nevertheless, the reported therapeutic efficacy of MSCs and EVs remains inconsistent across studies, underscoring the critical need for a deeper understanding of their immunomodulatory and paracrine mechanisms. This Research Topic therefore seeks to consolidate the latest research advances, offering a systematic exploration of pivotal breakthroughs and emerging directions in this rapidly evolving field.

Owing to their immunomodulatory and tissue-regenerative properties, MSCs hold considerable promise for treating autoimmune diseases such as systemic lupus erythematosus (SLE). However, their clinical translation has been hampered by persistent inconsistencies in efficacy—a consequence of heterogeneous cell origins, culture conditions, quality control, host immune dynamics, and concomitant immunosuppressive regimens. In this context, artificial intelligence (AI) is poised to revolutionize MSC-based therapy by systematically tackling these variables. As comprehensively reviewed by Kumar et al., AI can pinpoint optimal genetic and epigenetic targets, predict MSC behavior under diverse priming conditions, and tailor personalized treatment regimens. Beyond this, AI-driven analytics enable the refinement of dosing strategies and the rational integration of MSCs with immunosuppressants. By enhancing precision, consistency, and patient specificity, AI offers a transformative path forward—one that could markedly improve outcomes in SLE and, more broadly, propel the field toward genuinely patient-centered autoimmune care.

Yet the therapeutic potential of MSCs extends far beyond the cells themselves. Much attention has recently turned to their secreted EVs, which carry much of the paracrine cargo responsible for immunomodulation. In RA, Liu et al. have comprehensively summarized the evolving roles of EVs in disease progression, diagnosis, and therapeutic development, emphasizing that the next frontier lies in translating these discoveries into clinical applications for precision diagnosis and management.

More compelling is the opportunity for early intervention of RA. Since the pre-arthritic stage represents a critical window for preventing progression to full-blown RA, Shixiong W. et al. developed a robust murine model of pre-RA and evaluated MSC-derived microvesicles (MSC-MVs) as a prophylactic strategy. Their results are striking: treatment with MSC-MVs successfully reversed pathological features, underscoring their potential to fill a significant unmet need in the early management of RA.

Complementing these efforts, Wu et al. have synthesized recent advances in the regulatory roles of exosomal non-coding RNAs (ncRNAs) across a spectrum of autoimmune diseases—including SLE, RA, type 1 diabetes mellitus, inflammatory bowel disease, and Sjögren’s syndrome. They highlight the dual promise of exosomal ncRNAs as both diagnostic biomarkers and therapeutic targets, while candidly addressing the translational hurdles that remain: delivery efficiency, stability, immunogenicity, and the pressing need for large-scale validation. They propose a roadmap for integrating these insights into precision medicine strategies, paving the way for next-generation autoimmune therapies.

In addition to the application in treating autoimmune diseases, MSCs are increasingly recognized as promising candidates for treating tumors. MSCs are an important component of the tumor microenvironment (TME) and play a key role in tumor progression. However, the mechanism is unclear. Wang et al. conducted a meta-analysis review to investigate the role of MSCs in the EMT of head and neck squamous cell carcinoma (HNSCC) and its related mechanisms. Their results indicate that MSC intervention may promote EMT *in vitro*, as MSCs can reduce the expression of epithelial markers and increase mesenchymal markers and related transcription factors in cancer cells. Furthermore, the underlying molecular mechanisms suggest that this process may regulate multiple signaling pathways, such as NF-κB, PI3K/Akt/mTOR, IL-6R/JAK/STAT3. Collectively, these observations suggest MSCs may potentially promote EMT in HNSCC, thereby modulating tumor progression through multiple signaling pathway networks and providing new potential targets for future therapies targeting the TME.

Recently, MSCs have emerged as promising tumor-targeted delivery vehicles, leveraging their innate ability to sense inflammatory cues and home to tumor-adjacent stromal niches. Yu et al. have comprehensively reviewed the strategies for engineering MSCs in cancer therapy, centering on the concept of “turning enemies into allies.” These strategies encompass engineering MSCs into programmable intratumoral factories and carriers for delivering defined anticancer payloads, as well as antigen-directed approaches to enhance delivery efficiency and precision-release systems to minimize off-target effects. Therefore, further optimization of MSC-based therapeutic strategies through advanced genetic engineering and combination therapies holds substantial promise for improving their clinical efficacy.

Beyond their role as delivery vehicles, MSCs in the TME also critically interact with cancer stem-like cells (CSLCs) to sustain tumor malignancy and therapeutic resistance. CSLCs are a special subpopulation of cells within tumors that possess potent tumorigenic capacity, self-renewal, and differentiation potential. They are considered to be one of the fundamental causes driving tumor initiation, metastasis, recurrence, and therapeutic resistance. Targeting CSLCs has emerged as a novel therapeutic strategy for cancer. Sreekumar et al. reported that, in hepatocellular carcinoma (HCC), the triggering receptor expressed on myeloid cells 1 (TREM1) as a critical tumor-intrinsic regulator of liver cancer stem-like cells (LCSLCs) survival and tumorigenic potential, independent of its known immunomodulatory role in the TME. These findings suggest that targeting TREM1 may represent a promising dual-action therapeutic strategy for suppressing both cancer stem-like cell function and the pro-inflammatory tumor milieu in HCC.

In summary, this Research Topic underscores both the immense promise and the formidable challenges associated with MSC- and EV-based therapies for autoimmune diseases and cancer. Spanning AI-driven precision medicine, the emerging utility of EVs as diagnostic and prognostic biomarkers, and the bidirectional modulation and engineering of MSCs in oncological contexts. Future breakthroughs, however, will hinge critically on interdisciplinary collaboration, the establishment of standardized protocols, and the execution of large-scale, rigorously designed clinical trials. Ultimately, it is only through mechanism-guided precision and strategic innovation that the full therapeutic potential of MSCs and EVs can be translated into tangible clinical benefit for patients, thereby propelling regenerative medicine toward a new era of transformative impact.

